# Supplementary material for: Transcriptomic Insights into Mechanisms of Early Seed Maturation in the Garden Pea (Pisum sativum L.)
Source: Cells. 2020 Mar 23;9(3):779. doi: 10.3390/cells9030779 (PMC7140803; doi:10.3390/cells9030779)

Zhewan−1(25)

Global Summary

%DE = 0.86

genes with fdr < 0.2 = 1751 (634 + / 1117 −)

genes with fdr < 0.1 = 1630 (565 + / 1065 −)

genes with fdr < 0.05 = 1528 (511 + / 1017 −)

genes with fdr < 0.01 = 1195 (314 + / 881 −)

genes in genesets = 0

<FC> = 0

<t−score> = −27281.01

<p−value> = 0

<fdr> = 0.14

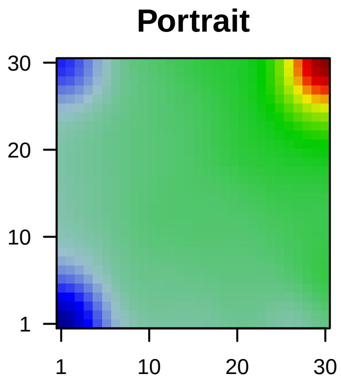

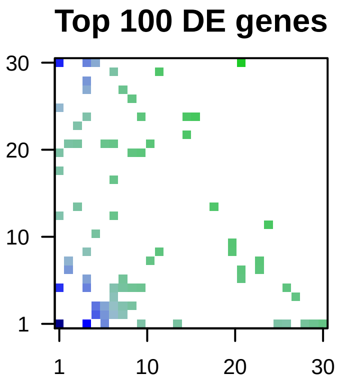

Global Genelist

Rank	ID	log(FC)	p−value	fdr	Metagene	Description
1	TRINITY_DN2	−0.85	0e+00	3e−14	1 x 1	
2	comp32560_c	−0.11	0e+00	3e−14	4 x 6	
3	comp40944_c	−0.02	0e+00	3e−14	16 x 24	
4	comp41855_c	−0.19	0e+00	3e−14	1 x 5	
5	comp46799_c	−0.01	0e+00	3e−14	9 x 20	
6	comp24437_c	−0.1	2e−16	3e−14	12 x 29	
7	comp31500_c	−0.07	2e−16	3e−14	7 x 3	
8	comp22509_c	−0.06	4e−16	4e−13	8 x 2	
9	comp41910_c	−0.04	2e−15	4e−13	9 x 5	
10	comp24517_c	−0.02	3e−15	7e−13	11 x 8	
11	comp6342_c0	−0.26	6e−15	7e−13	4 x 1	
12	comp20228_c	−0.03	8e−15	2e−12	10 x 5	
13	TRINITY_DN7	−0.86	2e−14	2e−12	1 x 1	
14	comp43362_c	−0.07	3e−14	2e−12	7 x 4	
15	TRINITY_DN1	−0.13	3e−14	3e−12	6 x 1	
16	comp19189_c	−0.01	4e−14	3e−12	9 x 20	
17	TRINITY_DN1	−0.75	5e−14	3e−12	1 x 1	
18	comp20491_c	−0.01	6e−14	2e−11	15 x 22	
19	TRINITY_DN2	−1.15	2e−13	2e−11	1 x 1	
20	comp11606_c	−0.05	2e−13	2e−11	6 x 21	

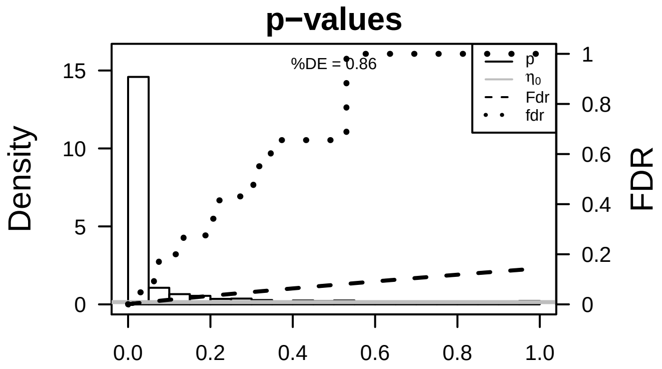

Supplement: Supplementary file 1 [file cells-09-00779-s001.zip › Supplementary materials/FileS4.zip!/FileS3/TFs - Results/Summary Sheets - Groups/Reports/Zhewan.1.25..pdf]
